# Glanzmann Thrombasthenia in a Newborn Due to a Rare Homozygous Missense Mutation

**DOI:** 10.7759/cureus.75291

**Published:** 2024-12-07

**Authors:** Saima Faraz, Fareeda Nikhat, Hiyam Hayel Suleiman Beshtawi, Sofia A Malik, Kauthar Yahya Hashim

**Affiliations:** 1 Obstetrics and Gynecology, Latifa Women and Children Hospital, Dubai, ARE; 2 Obstetrics and Gynecology, Latifa Hospital, Dubai, ARE

**Keywords:** consangunity, glanzmann thrombasthenia (gt), itgb3 gene defect, missense mutation, platelet function disorders

## Abstract

Glanzmann thrombasthenia (GT) is an autosomal recessive platelet functional bleeding disorder caused by mutations in the ITGA2B or ITGB3 genes, often presenting as mucocutaneous bleeding. GT typically presents in infancy, but this study reports a rare case of neonatal presentation in a female infant born to consanguineous parents. The mother, a 27-year-old woman with a family history of GT, presented at 36 weeks gestation for an elective cesarean due to a breech presentation. The newborn, delivered with an appearance, pulse, grimace, activity, and respiration (APGAR) score of 9, exhibited spontaneous bruising, gum bleeding, and hyperbilirubinemia, necessitating neonatal intensive care admission. An initial hematologic workup showed normal coagulation profiles, but platelet function was significantly impaired. Genetic analysis identified a homozygous ITGB3 mutation, p.Asp145Asn, with both parents confirmed as heterozygous carriers. Management included factor VIIa, platelet transfusions, fresh frozen plasma, and RBCs. This case underscores the critical need for early recognition of GT in neonates with severe bleeding, especially with family history and consanguinity, and highlights the implications of the p.Asp145Asn mutation in the severe neonatal presentation. Genetic counseling is recommended for the family given the 25% recurrence risk in future pregnancies, and prospective partner testing may aid in assessing recurrence risks for descendants.

## Introduction

Glanzmann thrombasthenia (GT) is a rare autosomal recessive bleeding disorder caused by a deficiency of the platelet integrin alpha IIb beta3, which is essential for platelet aggregation and hemostasis [1]. This deficiency disrupts normal clot formation, leading to recurrent mucocutaneous bleeding episodes. The condition is associated with mutations in the ITGA2B or ITGB3 genes on chromosome 17, with these genetic changes altering the function or expression of the integrin. GT’s prevalence is approximately 1 in 1,000,000, but it is significantly higher in populations with high rates of consanguinity [2].

Diagnosing GT involves ruling out more common bleeding disorders through a comprehensive approach, including a complete blood count, coagulation studies, and von Willebrand factor assays. Definitive diagnosis requires advanced platelet function tests and genetic analysis. Treatment follows a tiered strategy: mild bleeding episodes are managed with local hemostatic measures, whereas severe bleeding may require platelet transfusions or recombinant activated clotting factor VIIa (rFVIIa) [3].

Consanguineous marriages increase the risk of autosomal recessive disorders like GT because related couples are more likely to share and transmit the same gene variants. Research indicates that congenital anomalies are nearly twice as common among offspring of consanguineous unions. In populations with prevalent inbreeding, ancestral gene variants significantly influence the occurrence of these disorders. This case illustrates the impact of consanguinity, given that our patient exhibited symptoms such as bruising and gum bleeding shortly after birth rather than the typical presentation within the first year.

## Case presentation

**Figure 1:**
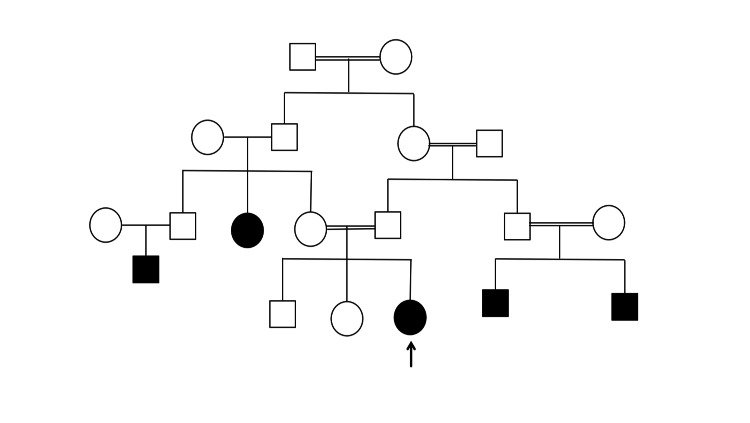
Pedigree of newborn female with GT (black arrow). The shaded shapes indicate clinically manifested patients of GT in the family (double line indicates consanguineous marriage) GT: Glanzmann thrombasthenia

A 27-year-old female with a first-degree consanguineous marriage presented at the 36th week of pregnancy with a breech presentation. Her previous two pregnancies were uneventful, resulting in spontaneous vaginal deliveries. Apart from a few sporadic episodes of epistaxis, her medical history was unremarkable. However, her family history was notable for GT because her sister had been diagnosed with the disease. Similarly, her husband’s siblings also had a history of GT, as shown in the pedigree in Figure 1. Because of the breech presentation, an elective cesarean section was planned.

On admission, her routine investigations revealed hypochromic microcytic anemia, whereas the rest of her biochemical and coagulation profiles were within normal limits. During the lower-segment cesarean section, she experienced significant postoperative vaginal bleeding. The baby, a girl weighing 2.9 kg with an appearance, pulse, grimace, activity, and respiration (APGAR) score of 9, presented with spontaneous skin bruising, gum bleeding, and hyperbilirubinemia. The rest of the neonatal checkup was normal. She was promptly transferred to the neonatal intensive care unit (NICU), intubated, and started on feeds on the second day of life. She was successfully weaned off ventilation by the sixth day.

Given the persistent oral bleeding, generalized bruising, and petechiae following birth, coupled with the breech delivery, a coordinated care plan was implemented in collaboration with the hematology team. Hematological investigations, including FBC, PT, APTT, TT, and fibrinogen levels, were unremarkable; however, platelet function assays showed severely reduced activity. Considering the strong family history of GT, the patient was empirically treated with recombinant factor VIIa (four doses in the first 24 hours), along with three units of platelets, one unit of fresh frozen plasma, and one packed RBC within the first 24 hours.

Genetic testing for the ITGB3 gene mutation confirmed a homozygous variant, p.Asp145Asn, consistent with GT type 2 (GT2). Four other family members were also identified as positive for the mutation. Amino acid and homocysteine levels were normal. Both parents were found to be heterozygous carriers of the mutation.

The infant was discharged on the seventh day of life and remains under regular follow-up with a hematologist and pediatrician.

## Discussion

This case highlights an unusual early presentation of GT at birth, a rare autosomal recessive bleeding disorder caused by mutations in the ITGA2B or ITGB3 genes [4]. Although GT typically manifests as mucocutaneous bleeding within the first year of life, this case demonstrated symptoms immediately after birth, including spontaneous skin bruising, gum bleeding, and hyperbilirubinemia, indicative of severe hemostatic impairment from birth [5]. This atypical presentation is attributed to the homozygous missense variant p.Asp145Asn in the ITGB3 gene, which encodes the integrin beta-3 subunit essential for platelet aggregation [6].

The ITGB3 variant, p.Asp145Asn, has been previously reported in individuals with GT and is associated with significant functional deficits in platelet integrin, leading to impaired platelet aggregation and defective clot retraction. The rarity of this variant is supported by its absence in large population databases such as the Genome Aggregation Database (gnomAD) [7]. Computational analyses and conservation studies suggest a substantial impact on protein function. Furthermore, another mutation at the same locus, p.Asp145Tyr, is classified as pathogenic, reinforcing the critical nature of this site for integrin beta-3 function [8].

In this case, early diagnosis was facilitated by a strong family history of GT alongside the neonate’s immediate clinical presentation. Laboratory evaluations revealed normal coagulation parameters but a markedly reduced platelet function assay, confirming the GT diagnosis. Genetic testing further verified the homozygous ITGB3 mutation in the neonate, with both parents identified as heterozygous carriers, consistent with the autosomal recessive inheritance pattern. The family’s consanguinity likely contributed to this genotype, aligning with increased GT prevalence in populations with high rates of consanguineous marriage.

Management required coordinated care from the hematology and neonatal teams. Because of persistent bleeding, the neonate received prompt treatment with recombinant factor VIIa, platelet transfusions, fresh frozen plasma, and red blood cell transfusions. This case underscores the necessity of immediate and specialized care for neonatal bleeding associated with GT because delays can be life-threatening.

Considering rare genetic bleeding disorders like GT is crucial in neonates with severe bleeding, particularly when consanguinity and relevant family history are evident. Genetic counseling for the family is essential because of the 25% recurrence risk in subsequent pregnancies. Carrier testing for potential reproductive partners is recommended to assess recurrence risks in future generations. Further research on the functional effects of the p.Asp145Asn mutation could enhance the understanding of genotype-phenotype correlations in GT to inform better clinical management and prognosis.

## Conclusions

This case highlights the importance of recognizing GT as a potential cause of severe neonatal bleeding, particularly in populations with high consanguinity rates and a relevant family history. The immediate presentation of GT in this neonate highlights the severe hemostatic impairment caused by the rare homozygous ITGB3 mutation, p.Asp145Asn, emphasizing its pathogenic significance.

Early diagnosis, facilitated by genetic testing and platelet function assays, is essential for timely management. Coordinated care, including factor VIIa, platelet transfusions, and other supportive measures, proved lifesaving in this case. Furthermore, this case emphasizes the necessity of genetic counseling for families with consanguineous marriages to mitigate the risk of recurrence.
